# Predicting Flavonoid UGT Regioselectivity

**DOI:** 10.1155/2011/506583

**Published:** 2011-06-30

**Authors:** Rhydon Jackson, Debra Knisley, Cecilia McIntosh, Phillip Pfeiffer

**Affiliations:** ^1^Technology Division, Momentx Corporation, Plano, TX 75024-3106, USA; ^2^Department of Mathematics and Statistics, East Tennessee State University, Johnson City, TN 37614, USA; ^3^Department of Biological Sciences, East Tennessee State University, Johnson City, TN 37614, USA; ^4^Department of Computer and Information Sciences, East Tennessee State University, Johnson City, TN 37614, USA

## Abstract

Machine learning was applied to a challenging and biologically significant protein classification problem: the prediction of avonoid UGT acceptor regioselectivity from primary sequence. Novel indices characterizing graphical models of residues were proposed and found to be widely distributed among existing amino acid indices and to cluster residues appropriately. UGT subsequences biochemically linked to regioselectivity were modeled as sets of index sequences. Several learning techniques incorporating these UGT models were compared with classifications based on standard sequence alignment scores. These techniques included an application of time series distance functions to protein classification. Time series distances defined on the index sequences were used in nearest neighbor and support vector machine classifiers. Additionally, Bayesian neural network classifiers were applied to the index sequences. The experiments identified improvements over the nearest neighbor and support vector machine classifications relying on standard alignment similarity scores, as well as strong correlations between specific subsequences and regioselectivities.

## 1. Introduction

This work was concerned with classifying of a set of closely related proteins, according to relatively finely scaled functional differences among them. These proteins are members of a subclass of uridine diphosphate glycosyltransferases (UGTs) known as flavonoid UGTS. Flavonoid UGTs are used by plants to help synthesize flavonoids, a class of compounds that are critical to a wide range of biological phenomena. The specific contribution to the synthesis process by the enzymes studied here is called glucosylation, that is, the addition of a sugar group to an emerging biomolecular structure. Glucosylation refers specifically to the attachment of a glucose sugar group. The general term for any sugar group is glycosylation. Glucosylation requires enzymes called glycosyltransferases (GTs) as catalysts. UGTs facilitate glycosylation from a donor called uridine diphosphate glucose. UGTs are extremely common among all organisms. General surveys of GTs include [1, 2], while [3] and more recently [4] focus on plant UGTs. 

Interest in flavonoid glycosylation arises due to flavonoids' medical and commercial benefits. Examples of medicinal benefits under investigation include reducing the incidence of cancer and heart disease, as well as anti-inflammatory activity [5, 6]. Some researchers link these benefits to flavonoid influence on signaling pathways affecting cellular function [7]. Flavonoids appear to mediate many defenses against environmental hazards [8]. Additionally, flavonoids are critical to interorganism signaling between plants and other organisms, for example, between roots and symbiotic bacteria in nitrogen fixation [9, 10]. Potential agricultural uses include expanding crop habitat through bioengineered nitrogen fixation [11]. Interest in flavonoid glycosylation also arises from an interest in glycosylation itself. Because glycosylation increases a molecule's availability and stability, it is extremely common in organisms and GTs are among the most numerous enzymes. [1–3] They are highly studied for pharmaceutical and other purposes. [12–16].

This study is concerned with *regioselectivity*, an important specialization exhibited by many flavonoid UGTs. During glycosylation, an acceptor may present multiple binding sites to the incoming sugar. Enzymes that exhibit regioselectivity, however, exhibit significant catalytic activity only at specific binding sites on the acceptor.

This work explores machine learning methods for predicting regioselectivity from flavonoid UGT sequence because understanding regioselectivity at the residue level has been frustrated by standard alignment methods. A mechanism for inferring flavonoid UGT regioselectivity directly from sequence could help direct biochemical analysis of glycosylation in flavonoid synthesis and facilitate the broader use of glycosylation in bioengineering. However, UGT regioselectivity has proved difficult to understand in terms of primary sequence. This difficulty stems from at least three sources. First, conformational cues from sequence are not helpful because the few structural determinations to date indicate that flavonoid UGTs have similar conformations despite often highly divergent sequences. Relatively few GTs and very few flavonoid UGTs have had their structures determined to date [4, 17]. The work of structural determination has been slowed by technical obstacles in generating sufficiently large GT quantities and in purifying and crystallizing them [18, 19]. This suggests that functional differences likely stem from small-scale variations at analogous loop regions bordering a common active site rather than large-scale conformational variations. [4, 17, 20, 21]. Secondly, in many cases divergent sequences exhibit uniform regioselectivity [22]. For example, [22] notes that sequence similarity varies from roughly 20% to 70% among 11 flavonoid UGTs in one plant, despite identical regioselectivity for a certain acceptor. Finally, in some cases, regioselectivity is a function of substrate. [21]. As one investigator summarizes, “The relationship between primary amino acid sequence, substrate specificity and product regioselectivity of plant UGTs is complex and remains to be determined.” [21].

In addition to the inherent interest of the domain, predicting flavonoid UGT regioselectivity is also an example of fine functional classification among closely related proteins. Such small-scale distinctions have not received wide attention in applications of machine learning to protein classification. Most applications of machine learning to protein classification from sequence have targeted broader classes like the SCOP fold, superfamily, or family level. In contrast, the two UGTs with known structures studied here have been placed in the same SCOP family. The classification derives from experimentally assayed biochemical behavior, not structural or primary sequence characteristics. Some recent work has applied machine learning toward phylogenomic identification of subfamily [23]. However, like regioselectivity, relevant classes do not always correspond to phylogenetic distinctions. For example, subfamilies of G protein-coupled receptors (GPCRs) share broad structural similarities, but cannot generally be distinguished by sequence or homology. One effort applying machine learning to GPCR subfamily classification is described in [24]. Other examples can be seen in any enzymatic case similar to UGTs, where subtle behavioral differences across structurally similar yet sequentially divergent and possibly phylogenetically divergent enzymes appear to be influenced by small differences in loop regions influencing to the active site. NB: need more on “small-scale not widely researched” angle.

Predicting flavonoid UGT regioselectivity presents a challenge that may be typical for other applications of machine learning to fine functional classification where standard sequence alignment cannot be relied on. A key component of the current work is to incorporate the biochemical findings that small-scale differences at key loop regions within similar structures appear to influence regioselectivity. Constructing feature vectors based on the loop regions is challenging. There are several such loop regions, and each of them presents relatively short sequences whose lengths vary across the data set of UGTs.

This work identifies promising techniques for addressing this challenge and compares several classification methods on the resulting feature spaces. Results are compared to *k*-nearest neighbor (*k*NN) classification using alignment score distances. The methods introduced here generally improve on classifiers using sequence alignment scores. In addition, one technique facilitated a focus on specific combinations of loop regions, allowing it to indicate that some loops under investigation may be particularly influential for the regioselectivity classes considered. When combined with biochemical data, these correlations could prove valuable in identifying mechanisms behind regioselectivity. 

## 2. Data

The data explored in this work consists of 23 UGTs. These 23 UGTs represent most of those whose regioselectivity has been biochemically verified [25, 26]. They vary in length between 446 and 511 residues. The data set is listed in Table 1.

Each UGT is represented by a row in the table. In order from left to right: the columns list a numeric identifier, an abbreviated label, the primary kind of substrate that accepts glucosylation for the UGT, the UGT's native organism, an accession number, and the UGT's regioselectivity class. A primed regioselectivity class label is distinct from an unprimed one, e. UGT 23 is not in the 3-O class. See [26] for more detail on the structural distinctions among substrate acceptor positions. Following the table, this work refers to UGTs by numeric ID or the label. Most labels encode a UGT's organism, acceptor type, and acceptor position class, in that order. Two exceptions are made for UGTs 3 and 23, which are labeled with names previously established in the literature.

With one exception, previously published biochemical characterizations are available. Sequence characterizations and details are taken from the following sources respectively: 1, [27]; 2, [28]; 3, [17]; 4, [29]; 5–7, [30]; 8, [31]; 9, [32]; 10, [33]; 11, [34]; 12-13, [35]; 14, [36]; 15-16, [37]; 17, [38]; 18, [39]; 19, [40]; 20, [41]; 21-22, [42]; 23, [20]. The class for CsF3GT is assumed because a closely related UGT is regioselective for the 3-O position. [19] All but one glycosylate flavonoid substrates. Two that normally glycosylate nonflavonoid substrates, starred in the table, have also been shown to act on flavonoids. CuLGT glycosylates a limonoid substrate that is larger and less polar than flavonoids, but it is not an extreme alignment outlier. Including it provided a data point outside the target classes for substantially different reasons than the other negative examples.

**Figure 1:**
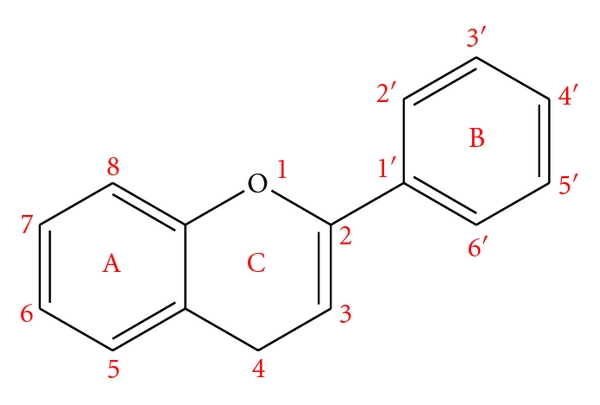
Common Flavonoid Carbon Skeleton Structure. Three rings labeled A, B, and C are arranged in a fixed pattern. One vertex in the central ring labeled C is occupied by an oxygen atom rather than carbon. Each vertex is given a label, running from 1 through 8 for the adjacent rings A and C, while ranging from 1′ through 6′ on the offset ring B.

The study explores preferences for two binding positions, known as the 3-O and the 7-O positions.  Figure 1 illustrates a common flavonoid carbon skeleton structure. Flavonoids are characterized by the appearance of various functional groups on these vertices. For example, the flavonoid kaempferol has hydroxyl groups at vertices 3, 5, 7, and 4′; hydrogen atoms at vertices 3′ and 5′; and a double-bonded oxygen atom at vertex 4.

Flavonoid glycosylation typically occurs at ring positions bearing hydroxyl groups. More flavonoid subclasses bear a hydroxyl group at the 7 position than at the 3 position. Reference [43] diagrams the basic skeletons for each flavonoid subtype. Similarly, among flavonoid UGTs that are promiscuous with acceptor subtype, 7-O UGTs might range over more subtypes than 3-O UGTs. Thus, it is reasonable to expect that 7-O flavonoid UGTs may be more difficult to classify than 3-O UGTs because there is a greater substrate variety for those UGTs to cover.

3-O and the 7-O regioselectivities have higher frequencies among the data set, 10 and 6, respectively, than others. Given the small data set, a two-class approach instead of a multiclass approach is taken, that is, each classification algorithm was applied twice, once using the two classes 3-O and not-3-O and then using the classes 7-O and not-7-O, because this allowed larger class populations than would be the case in multiclass training. Pairwise identity variation is large among the classes. For example, the 3-O and not-3-O variations are 22%–51% and 27%–70%, respectively. 

## 3. Methods

### 3.1. Residue Models

This work is based on representing amino acid residues numerically. Novel amino acid indices derived from the structural properties of individual amino acids are introduced. The indices are graph-theoretic-based measures that are sensitive to small changes in structure.

At least three considerations suggest the use of graphical models to characterize residues. First, the field of graph theory is ripe with invariant measures that characterize structural properties of the graph. The application of graphical models of molecular structure is well established in the study of quantitative structure-activity relationships (QSARs) [44, 45]. However, the use of graphs to characterize amino acid residues descriptors is largely an unexplored resource. Moreover, side-chains are well suited to description via rooted graphs. Lastly, recent work indicates that techniques from graph theory not widely applied to QSAR can capture significant structural qualities. For example, tree models of natural RNA secondary structure are characterized by descriptors stemming from domination theory, [46].

**Figure 2:**
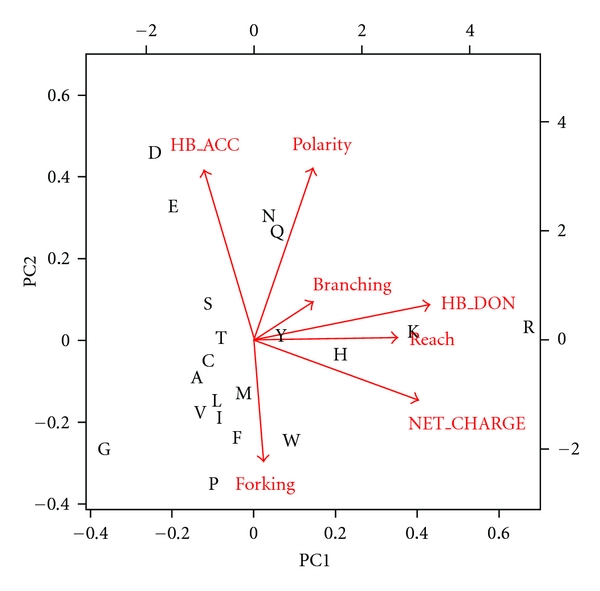
Biplot of Residue Index PCA. The space spanned by the first two principal components of the seven raw indices is shown at two scales. The location of each residue type is given by the left and bottom scales and indicated by a single-character abbreviation. Projections of the original basis vectors from the raw indices are shown as vectors with components given by the top and right scales.

Each side-chain was modeled as a graph where atoms, including the *α*-carbon, are treated as nodes and bonds as edges. The second bond between proline's side-chain and the backbone was also included. The graphs' elements were labeled with relevant physical quantities and used to derive 7 numerical indices incorporating these quantities and side-chain topology. The residue indices, which exhibited some strong covariance, were recast using principal component analysis (PCA). The indices are defined as follows: 

After refinement by PCA, the indices were found to cluster residues in a reasonable way.  Figure 2 displays a biplot of each residue in the space spanned by the first two principal components. Note that the aliphatic residues I, V, and L are grouped tightly by PC1 and PC2. The positively charged residues H, K, and R are clearly separated as a class by high PC1 values. The acidic residues D and E are isolated with high PC2 values and low PC1 values.

The indices are also well distributed among the existing indices. Using the software and data described by [48], the original raw indices and the PCA refined set were plotted in a minimum spanning tree (MST) including over 460 other established amino acid indices. These established indices have been devised from various considerations such as physicochemical features and evolutionary substitution rates. The refined PCA indices are generally well separated, appearing in several distinct neighborhoods in the spanning tree. See [26] for a table of raw index values, the PCA refinements, and more details on the distribution of indices on the spanning tree. 

#### 3.1.1. Reach

The graph's diameter, measuring all distances from the *α*-carbon and taking bond length as edge weight. Reach measures a side-chain's size.

#### 3.1.2. Branching Density

The average degree of the graph's internal nodes, that is, those of degree two or more. Branching density measures the leafiness of a side-chain's graph model.

#### 3.1.3. Forking Index

Using unit edge weights, the number of internal nodes at each distance from the *α*-carbon is weighted by the reciprocal of the distance and then tallied. This measures the presence of lengthy branches in a side-chain.

#### 3.1.4. Net Partial Charge

The sum of partial charges for each atom in a residue.

#### 3.1.5. Average Polarity

A simple average of edge weights where weights are given by the difference in partial charge between adjacent nodes multiplied by bond distance, that is, an average of the bond dipoles within a side-chain.

#### 3.1.6. HB Acceptor Index

A weighted tally of lone electron pairs on N, O, or S atoms known to accept hydrogen bonds. Atoms for which the survey in [47] indicates no appreciable hydrogen bonding are ignored in this index and the following one. The terms are weighted by the electronegativity of the atom bearing the pair and bond distance from the *α*-carbon. This measures a residue's tendency to accept hydrogen bonds.

#### 3.1.7. HB Donor Index

A weighted tally of H atoms known to donate hydrogen bonds and bound to N, O, or S atoms. The terms are weighted by electronegativity and bond distance from the N, O, or S atom to the *α*-carbon. Donor index measures a residue's tendency to donate to hydrogen bonds.

### 3.2. Loop Regions

Loop regions linked to regioselectivity were identified using published analyses of experimentally determined UGT structures and biochemical characterizations of UGT activity. These analyses indicate that the catalytic action of these UGTs occurs inside the cleft between the two Rossmann-fold domains comprising these proteins, largely formed by seven analogous loop regions in each UGT. There is a consensus that enzymatic interaction with acceptors is generally connected with these seven loop regions, although not exclusively so [4, 17, 20, 21, 49, 50]. The seven loop regions forming this *acceptor pocket* are as follows: 

 (a) *Nβ*1 − *Nα*1,

(b) *Nβ*2 − *Nα*2,

(c) *Nβ*3 − *Nα*3,

(d) *Nβ*4 − *Nα*4,

(e) *Nβ*5*a* − *Nα*5*a*,

(f) *Cβ*1 − *Cα*1,

(g) *Cβ*5 − *Cα*5.

The experimentally determined structures available for sequences VvGT and UGT71G1 were used to determine their loops. Because the other sequences lack experimentally determined structures, putative loop identifications were obtained from secondary structure prediction algorithms. This is a problem with GPCRs too. Likewise, here again prediction mechanisms exist for extracellular and intracellular regions, should they be a focus of classification. Secondary structure predictions from two sources were compared [51, 52]. When available, a consensus was used. Other cases were judged individually, based on the two prediction results, the propensity of individual residue types for either alpha helix or beta strand inclusion, and alignment with the two known structures. An effort was made to err on the side of longer putative loops. One conserved histidine present in the first loop of every UGT was ignored since its presence was identical in every case. See [26] for a specification of the loop regions in terms of residue position. 

The seven key loops were taken as the basis for the machine learning techniques applied to regioselectivity in this work. Although it appeared reasonable to treat these loops as significant for interaction with the acceptor, this assumption is subject to six caveats: (1) the acceptor pocket can include portions of non-loop regions; (2) enzyme characteristics like regioselectivity depend on acceptor structure as well as enzyme structure; (3) catalytic activity varies in its dependence on conserved residues among the UGTs considered here; (4) mutagenic studies have demonstrated that in some cases residues lying outside of these loops can be necessary, as opposed to sufficient, to regioselectivity because they impact tertiary structural qualities such as the size of the cleft between a UGT's two domains; (5) some sequence patterns that yield excellent predictors for some regioselectivities are difficult to connect directly to the acceptor pocket lining, for example, a full-sequence alignment of the UGTs considered here reveals that the E and S residues at positions 288 and 306 on VvGT are good predictors of 3-O regioselectivity among this set of UGTs, despite being remote from the acceptor pocket; see [26] for more details; and (6) the speculative nature of the loop identification via secondary structure prediction may be a source of some error. While these caveats should inform the results described here, the putative loops are considered a reasonable basis for an initial exploration of regioselectivity classification by machine learning.

The loop regions are represented as numerical sequences of index values. Thus, each residue within a loop is represented by a vector of its index values. A loop yields a sequence of index vectors. Finally, a UGT yields the set of vector sequences representing its loops. In this way, each UGT is mapped to a set of multidimensional time series.

The loop regions appear within the UGTs as short, isolated loop subsequences of varying length. They can be as short as one residue, and roughly two-thirds are under six residues long. From UGT to UGT, the range of length variation in the seven loop regions is 3–6, 2–18, 7–24, 2-3, 1–4, 2–9, and 2–9, respectively. This variation prevents direct classification methods like neural networks or support vector machines (SVMs) which expect input spaces with fixed dimensions. Similarly, distance based classifications like *k*NN are not immediately suitable either, since standard distances like the Euclidean measure also require input spaces with fixed dimensions.

This work takes two approaches to this problem. On the one hand, it attempts to derive regions of equal length from the unequal loop regions. This strategy identified for each region a set of uniformly long subsequences, one per UGT, such that each subsequence covered its loop on its UGT. Loop locations within these covering subsequences were regularized by first performing a gapless, local alignment across all 23 UGTs around each region. The aligned sequences were then cropped to obtain a uniform length while retaining the loop. See [26] for a specification of the covering subsequences in terms of residue position. Concatenating the covering subsequences for a UGT yielded sequences of identical length. After mapping the residue sequences to sequences of index vectors, flattening the vectors by concatenation transformed the UGT representations into uniform-length, one-dimensional numerical sequences.

**Figure 3:**
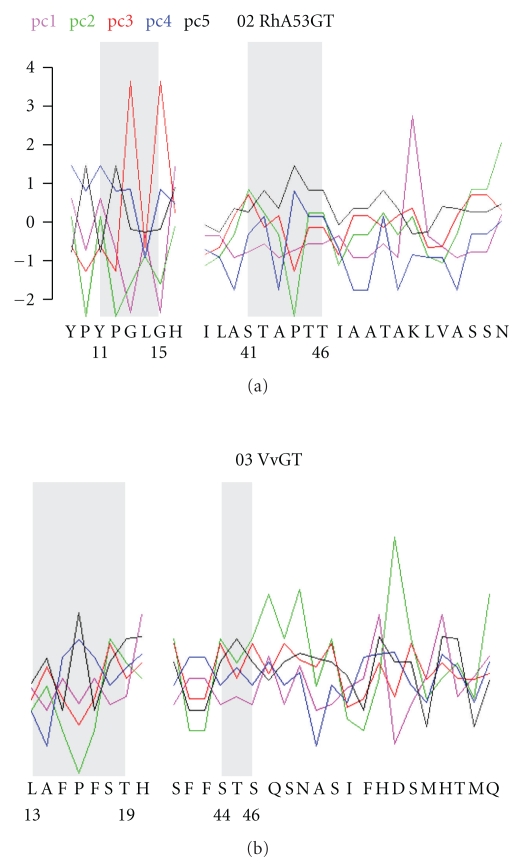
Residue Indices, Aligned Covering Subsequences, and Loop Regions. The covering subsequence for the first two loop regions is depicted for RhA53GT and VvGT. The letters running along a horizontal axis indicate the residue subsequence. Shaded backgrounds indicate the loop regions within each covering subsequence. The location of a loop region is indicated by numerals giving the beginning and ending residue positions within the full sequence. The first five PCA indices are plotted in contrasting colors for each residue. A common scale is provided in the top left corner.

Figure 3 illustrates the relation between covering subsequences and loop regions. While a loop region varies in length from case to case, covering subsequences do not. For example, the second loop region in VvGT has been identified as running from S-44 to S-56. However, a local alignment around the second loop region covering that region in all 23 UGTs wound up being significantly longer than the second loop region for VvGT. Hence, the covering subsequence for that loop runs from S-41 to Q-61 on VvGT.

The second approach avoided alignment and cropping by applying a distance function arising from work on time series classification to measure dissimilarity among bare loop sequences.

### 3.3. Distance Measures

Distance measures for time series has attracted extensive research [53–56]. Time series distance functions are commonly used in signal processing applications such as spoken language recognition, image matching, and video library searches. [56].

This work investigated three time series distance measures: dynamic time warping (DTW), longest common subsequence (LCSS), and minimum variance matching (MVM). These distances can be briefly described as follows.

Instead of aligning two time series side by side and comparing the distance between values at identical time points, DTW allows values at different time points to be matched. By introducing a distance cost for this time warping and minimizing the overall distance via dynamic programming, DTW yields a distance measure for time series that aggregates series differing mostly by time compression, dilation, or phase. DTW is asymmetric in that is matches every value in one of the compared sequences to some value in the second.

LCSS determines the longest common subsequence between two time series where commonality is taken to be numerical similarity relative to some threshold. In the version studied here, some time warping is also allowed. Given the length of a longest common subsequence, an LCSS similarity measure is obtained from the ratio of that length to that of the longer of the two original sequences. A distance measure was obtained by taking the difference between the LCSS score and unity. Because it is grounded on subsequence comparisons, LCSS can ignore values in both sequences.

MVM in a way combines these approaches by minimizing subsequence distances while allowing time warping. MVM exhibits the same kind of asymmetry as DTW.

The distance between corresponding loops on two UGTs can be taken as the sum of the distances between each index series over the loop. The distance between two UGTs can be calculated by summing the distances between their respective loops. Alternatively, any combination of loops can be isolated by restricting the sum to just those loops. Similarly, any combination of indices can be isolated by restricting the sum to just those indices.

In order to select one of these time series distance functions, they were ranked by their performance in *k*NN classifiers using the full distance sum including every loop and index. The values used for *k* were 1, 3, and 5. Performance was assessed with crossvalidation, using a leave-six-out and a leave-one-out approach. DTW was the best performer overall. An existing implementation was used for time series distance calculations [57].

Using time series distances to classify proteins is structurally quite similar to firmly established sequence alignment classifiers. While both involve sequence matching and rely on techniques like gap costs, time series distance functions compare bare numerical values rather than relying on a matching score provided to letters from a finite alphabet. In effect, using a time series distance function with residue indices enhances traditional sequence alignment methods by allowing more subtle matching behavior. In this respect, it is an alternative to specialized alignment score matrices.

Previous protein classifications have incorporated frequency analysis of time series models. For example, [58] investigated a classification method based on wavelets derived from residue index series. However, there appear to be few examples of using time series distance functions for protein classification. The sole example known to the authors was only recently published. [59].

Using time series distances for this work offers several benefits. First, it gives meaningful distances even for very short lengths. Additionally, it provides a way to handle unequally long loop regions. Finally, it offers a simple way to focus on specific combinations of loops and indices.

Although wavelets appear to offer a reasonable alternative to time series distances, the short loop regions of varying lengths prevent as straightforward an application of wavelets to distances as the time series distance measures allow. Wavelets allow a decomposition in terms of signal variation at different time scales and at different locations within a time series. However, the loop regions studied in the current work are frequently too short for this multiresolution analysis. For example, when comparing a series of length 1 to one of length 4, the scale of interest is one time unit. Moreover, any wavelet analysis in the current work would confront one of two problems. A distance measure for the decomposition must either blend information from all time locations, as in [58], or handle the fact that the locations of interest vary in length.

### 3.4. Classification Methods

This work investigates the performance of Bayesian neural networks (BNNs), support vector machines (SVMs), and *k*NN classifiers.

BNNs were applied to the one-dimensional feature vectors obtained from the uniform length covering subsequences. BNNs are artificial neural networks trained by a hierarchical method of Bayesian inference. [60–62] This learning technique treats network weights as a multidimensional random variable with some prior distribution. Applying Bayes' formula converts the prior to a posterior distribution based on the evidence provided in the training data. In other words, the probability of the weights given the data is expressed as a function of a prior probability of the weights and the probability of the data assuming that prior. The result is a full posterior distribution of weight values yielding a distribution of output values. This can be considered as a set of networks, each of whose significance is quantified by the posterior distribution. The posterior distribution can then be employed to obtain a single expectation value for the BNN as a whole.

The BNN classification relay an established implementation using a hybrid Markov Chain Monte Carlo (MCMC) technique for calculating predictive distributions [60]. Priors for the network parameters were determined by a hierarchical method. The networks were initialized to have zero-valued weights and biases, and each network parameter was given an independent, zero-mean Gaussian prior. The precision of these priors were specified in a group-wise fashion using four groups: input-to-hidden weights, hidden unit biases, hidden-to-output weights, and the output unit. All the Gaussians within a single group were given the same precision. Letting *τ* be the precision for a group of network parameters, the corresponding standard deviation is given by *σ* = *τ* ^−2^. Following a strategy used in [60], the Gaussian distribution for the output unit's bias was always given a fixed precision. Precisions for the other priors were either fixed or drawn from the joint hyperpriors given by Gamma distributions. The Gamma distributions were controlled by a pair of shape and scale hyperparameters. Decreasing the shape parameter for this prior tends to spread out the distribution making it more vague. The scale parameter can be taken as a rough approximation of the mean. Writing *α* and *w* for the shape and scale hyperparameters and *τ* for the precision, the implementation parameterizes Gamma distributions of precisions for a group as follows:

(1) $$\begin{array}{c} P\left(\tau \right)\;=\frac{{\left(\alpha /2w\right)}^{\alpha /2}}{\Gamma \left(\alpha /2\right)}\tau^{(\alpha /2)-1}e^{-\tau \alpha /2w} \end{array}.$$

This hierarchical Bayesian structure is one of the implementation's key features. It simultaneously achieves two significant benefits. First, it increases the efficiency of Bayesian inference by qualifying the hyperprior with observed data. For a simpler exposition, this paragraph will refer to both network parameters, that is, weights and biases, by the one term “weights”. The hierarchical structure allows the weight updates obtained by hybrid MCMC sampling to qualify the precisions of those weights' priors. As a result, the training consists of a series of alternating operation types: (a) hybrid MCMC operations that update the weights as a function of the priors and (b) hyperparameter operations that update the priors as a function of the weights. This empirical qualification of the priors is obtained by an application of Bayes' formula to an assumed joint distribution of the weights and the precision controlling the priors over the weights. More concretely, let the distribution of the weights in a group conditional on the common precision for their Gaussians be *P*(*w*_1_, *w*_2_,…, *w*_*k*_|*τ*). Then, updated weights can inform a new precision because *P*(*τw*_1_, *w*_2_,…, *w*_*k*_) ∝ *P*(*w*_1_, *w*_2_,…, *w*_*k*_|*τ*)*P*(*τ*), where *P*(*τ*) is the Gamma hyperprior. Because each group's hyperparameter can be updated one by one, this amounts to Gibbs sampling from the hyperprior. Second, the hierarchical structure also mitigates overfitting by introducing interdependencies among a large number of parameters. [63].

All BNN training efforts used a common network topology. The concatenated covering subsequences amount to 86 residues. Since each residue was mapped to 7 indices, the network included 86 × 7 = 602 inputs, with a single binary output. A simple topology of one hidden layer with 301 units was used. The layers were fully connected. The hidden units and output unit were supplied with independent biases, yielding a total of 181, 805 weights. While the ratio of weights to training size far exceeds the limits a standard rule of thumb would indicate for reasonable error [64], the hierarchical Bayesian approach might be expected to mitigate this concern [60, 63]. The units used *tanh* activation functions.

As suggested by [60], sampling for the BNN training efforts was usually constructed in two phases. A first, short sampling phase with a relatively low computational cost was used to move the system near equilibrium. Often this initial phase relied on fixed precisions for the network parameter Gaussians. A second, more patient sampling phase was then used to achieve equilibrium. The second phase typically updated the Gaussian precisions using the Gibbs sampling from the Gamma hyperpriors. As in [60], the primary criteria used to judge the arrival of equilibrium were the average squared error on the training set and the range of variation in hyperparameters and weights. Using this general framework, various specifications for priors and MCMC sampling runs were made on a trial and error basis. Trials consisted of selecting a prior specification and then trying a variety of sampling specifications in order to reach equilibrium.

SVMs classify by embedding training data in a different space whose properties facilitate the identification of optimal class boundaries [65, 66]. By using a reproducing kernel Hilbert space for the new feature space, SVMs facilitate the identification of optimal class boundaries through generic optimization techniques. In turn, these boundaries are used to estimate the classification of previously unseen data.

Distance or dissimilarity measures are a standard method of deriving kernels for machine learning [65, 67]. For example, a distance or dissimilarity function from an object to a reference point, say *x*_*O*_, can be used to define a kernel as follows: *k*_*O*_(*x*, *y*) = (1/2)(−*d* ^2^(*x*, *y*) + *d* ^2^(*x*, *x*_*O*_) + *d* ^2^(*y*, *x*_*O*_)) [68]. However, nonmetric distance functions like DTW cannot guarantee that the optimization step for SVMs will reach a true optimum. One of several approaches to coping with this issue [65, 67–70] uses a standard kernel with feature vectors derived from the distance measure. Such feature vectors can be readily obtained by representing an object as the vector of its distances from each member of a reference set [68]. That is the approach taken in this work. A feature vector was obtained for each UGT by taking as components each of the DTW distances from its sequence to those of a training set of UGTs.

Existing implementations were used for the SVMs [71]. A popular generic choice was used for a kernel function, the radial basis function. Kernel parameters were obtained from a heuristic method delivered with the implementation.

*k*NN classifiers were applied using the DTW distance measures. *k*NN classifiers use distance measures to classify by assigning an input to the most frequent class among *k* the nearest neighbors. Values of 1, 3 and 5 were investigated, with 3 having the best overall performance. Because it incurred very low training costs, 3NN classifiers were also applied to all combinations of loops and the first 5 indices.

### 3.5. Evaluation

Comparisons were made among the BNN classifiers and the SVM and 3NN classifiers based on DTW distances. These were also compared to 3NN and SVM classifiers using a simple distance measure derived from sequence alignment scores.

Although typically used in the guise of phylogenetic trees, nearest neighbor methods based on sequence alignment scores are a common tool in protein classification. The alignment classifications used a distance function obtained by specifying a simple decreasing function of scores. Alignment distance was measured using the reciprocal of the alignment score as computed by the second version of the ClustalW algorithm, using the implementation's default parameter values [72]. This algorithm uses a progressive alignment scheme iterating over a range of substitution matrices. The default parameter values used were those accompanying the implementation of the algorithm by its authors.

SVMs were trained using distances from full-sequence and loop region alignments. The subsequence alignment distances used in SVMs were obtained from alignments of concatenated loops. In contrast to the SVM subsequence alignment distances, the alignment distances used in 3NN classifiers were obtained by summing distances derived from aligning individual loop regions. This provided a loop combination specificity for the alignment distance 3NN classifiers, allowing comparison with the loop specific DTW 3NN classifiers.

This work requires care to avoid overfitting due to the small data set and complex feature spaces. Therefore, the evaluation of classifier performance in this work ignores training error, focusing exclusively on generalization performance as estimated by crossvalidation [61, 73]. The low computational cost of the SVM and most of the 3NN methods enabled crossvalidations with both a leave-six-out (L6O) and a leave-one-out (L1O) approach. An exception was made for the loop-index-specific 3NN classifiers, which were crossvalidated only with an L1O approach due to the high number of loop-index combinations. Training for the BNN took considerably longer than other methods and only an L6O approach was used for it.

The combination of a small data set and very regular error patterns in crossvalidations among all the classifiers and hold-out set sizes allows a direct comparison of errors in place of typical statistical measures of classifier performance such as areas under ROC curves. In other words, the misclassified UGTs tend to come from the same subset regardless of the classifier method and hold out set size, yet some methods miss fewer in this subset than others. In order to compare two classifiers, it suffices to see which missed fewer among the common set of problematic UGTs.

## 4. Results

 The results separate into two sets: those pertaining to 3-O regioselectivity and those pertaining to 7-O regioselectivity. Another division is between results obtained using all loops and indices and those from loop-index-specific (LIS) classifiers. The results obtained using all loops and indices will be discussed next, while the loop-index-specific results will discussed separately.

**Figure 4:**
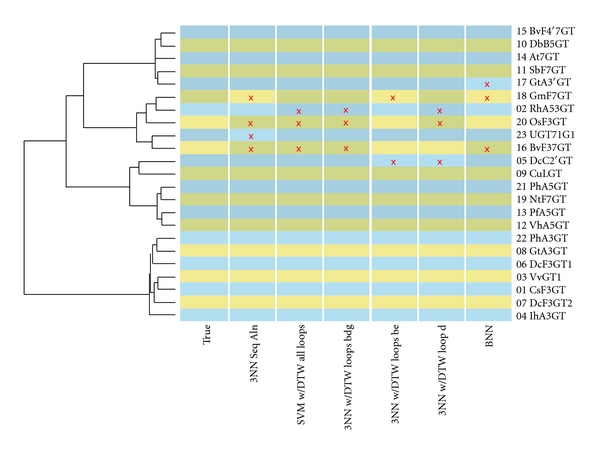
Experimental and Predicted 3-O Regioselectivity Classification. Each UGT is represented as a row and labeled at right. A dendrogram at left indicates phylogenetic relationships based on sequence alignment. Columns of colored cells, labeled underneath, indicate true classifications and those of selected methods under study. Positive and negative classifications are indicated by light and dark shades, respectively. Alternating hues distinguish adjacent rows. Red x's emphasize classification errors. (Alignment made with [72]).

The 3-O findings are summarized in Figure 4. Figure 4 shows a phylogenetic tree of the 23 UGTs investigated here based on full-sequence alignment scores. A heatmap panel indicates the true 3-O class for each UGT as well as classifications from comparably performing methods studied in this work.

The errors all come from a relatively small set comprised by 2, 5, 16, 17, 18, 20, and 23. These UGTs appear in mixed-class alignment clusters in Figure 4. This uniformity is reasonable because the UGTs that are regularly missed include several that have exhibited promiscuous regioselectivity and are also sequentially close. 3NN with full-sequence alignment scores misses 4 in a L1O crossvalidation and 5 with L6O. In other words, it misses most of the “hard cases.” SVM classification using the same alignment distances misses 5 or 6, depending on the hold out set size. Restricting the alignment to the covering subsequences offers no improvement. DTW distances do even worse in 3NN classifiers, missing the entire cluster. However, the DTW distances used in an SVM shrink the error set to 2, 16, and 20 whether the crossvalidation used is L1O or L6O. Finally, the BNN method also results in a small error set of 3: 16, 17, and 18. UGT 16 seems a particularly hard case, being missed by every method except the loop-specific classification discussed below. In summary, DTW SVMs and the BNNs offer small but notable improvements over classification by the traditional sequence alignment.

**Figure 5:**
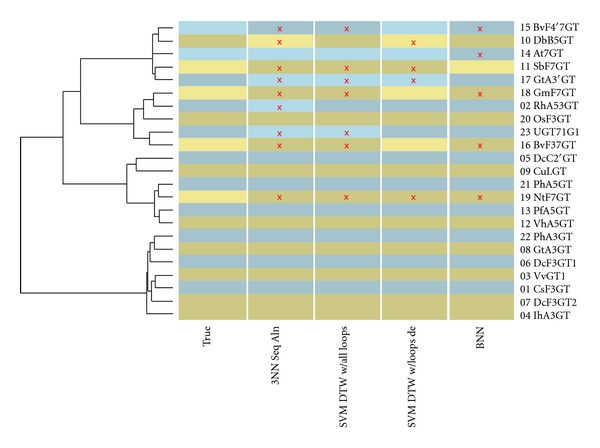
Experimental and Predicted 7-O Regioselectivity Classification. Each UGT is represented as a row and labeled at right. A dendrogram at left indicates phylogenetic relationships based on sequence alignment. Columns of colored cells, labeled underneath, indicate true classifications and those of selected methods under study. Positive and negative classifications are indicated by light and dark shades, respectively. Alternating hues distinguish adjacent rows. Red x's emphasize classification errors. (Alignment made with [72]).

In contrast to the 3-O problem, all methods had difficulty clustering UGTs by regioselectivity at the 7-O position. The error rates approach 50%, with false negative rates reaching 100%. For example, the L1O 3NN classifier relying on full-sequence alignment distances missed on UGTs 2, 10, 11, 15, 16, 17, 18, 19, and 23. The L1O 3NN DTW classifier missed these UGTs as well as 5. The SVM using the DTW distances did somewhat better, missing 11, 15, 16, 17, 18, 19, and 23. Like the 3-O results, errors tended to come from the same set. However, this set included all of the positive cases. BNNs trained while holding six UGTs out did only marginally better, missing on 14, 15, 16, 18, and 19 while correctly classifying the positive case 11. UGT 19, a local anomaly in a cluster of 5-O UGTs, was missed by all 7-O classifiers. The 7-O findings are summarized in Figure 5.

**Figure 6:**
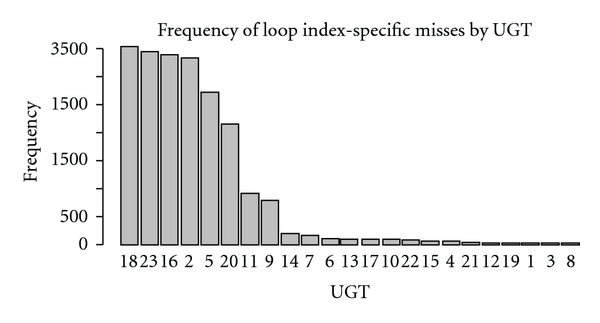
Frequency of Classification Errors within Loop-Index-Specific Classifiers by UGT. For each UGT, the number of loop-index-specific 3NN classifiers which incorrectly classified that UGT is plotted.

Turning to the loop-index-specific results on the 3-O problem, the success of the 3NN DTW methods using selected indices and loop regions ranged widely across the 3, 397 combinatorial possibilities. However, within this range, there was a great deal of regularity.  Figure 6 shows the distribution of the frequency of errors by individual UGT. By far the most frequently missed UGTs were 2, 5, 11, 16, 18, 20, and 23—all from the same problematic set indicated in Figure 4. The frequency of misses declines sharply from there. In fact, the most frequent five loss patterns were (2, 5, 16, 18, 20, 23), (2, 5, 16, 18, 23), (2, 5, 11, 16, 18, 23), (2, 16, 18, 20, 23), and (2, 5, 11, 16, 18, 20, 23) occurring with frequencies of 584, 312, 252, 246, and 243, respectively. The remaining 507 distinct loss patterns had sharply falling frequencies of less 200. Thus, most loop-index combinations performed poorly, missing all six of the UGTs in the mixed cluster of hard cases.

However, in several cases, exceptional performance was very highly correlated with the loop combination used. In a very small number of isolated cases, low loss could not be correlated with loop combination. It is assumed these are artifacts of estimating generalization error by crossvalidation on such a small data set. These high performing cases were identified by investigating the distribution of loss patterns within a given loop combination. Distributions characterized by a pronounced mode with a small loss relative to the sequence based 3NN loss of 4 were sought.

Table 2 lists the loss patterns for the three most accurate loop combinations. Loops are indicated by the letters a–g and an index by the numerals 1–5. False positives are starred. The most frequent loss pattern is always returned by the classifier using every index. As different indices are dropped out, the loss pattern varies somewhat but largely stays constant. Thus, the results using all indices were typical of its loop combination over all.

The highest performing loop combinations were d, be, and bdg. Only these three loop combinations presented modes with a loss under 4 and a frequency higher than 9, that is, a frequency covering roughly a third or more of the 31 loop-index choices for a given loop combination. Two other combinations had a single mode with a loss fewer than 4: loop combination bde missed UGTs 5, 18, and 23 a total of 8 times; abe missed UGTs 5, 18, and 23 a total of 9 times. In structural determinations to date, these three loops appear to be the closest to the acceptor among the seven key loops. See Figure 8 in [4].

The correlations in Table 2 are striking. This is especially so in the case of the extremely regular loss pattern for loop d. This data suggests that loop d is highly influential in the regioselectivity of the hard cases 16, 18, and 23, while not having as much influence on the regioselectivity of UGTs 2, 5, and 20. Similarly, there seems to be a strong connection between loops b and e and the regioselectivity of UGTs 2, 16, 20, and 23; but not UGTs 5 and 18. These findings also suggest a strong connection between loops b, d, and g the regioselectivity of UGTs 5, 18, and 23; but not 2, 16, and 20. Taken together these three loop combinations correctly classify all UGTs in the 3-O problem. However, a simple majority among the three loop combinations still misses 2, 5, and 20.

These classifications could not be obtained by focusing sequence alignment methods on the same loop combinations. Using a summed sequence distance in L1O 3NN classifiers led to loss patterns for the bdg, be, and d loop combinations of 2, 5, 9, 16, 20; 9 (UGT 9 was a frequent problem for the DTW *K*NN be classifier as well), 18; and 2, 5, 11, 15, 16, 21, 23, respectively. Alignment methods focused on the loop combination be yielded substantial improvements over full-sequence alignment classifiers. However, alignment alone could not uncover the other correlations in Table 2. It appears that the physical aspects modeled in the indices and the time series distance classification that brings them to bear were critical to discovering these correlations.

The *k*NN method using selected indices and loop regions also had some success on the 7-O problem, with the highest accuracy yielding classification errors on 3 cases: a 50% false negative rate.  Table 3 lists these loop-index combinations. False positives are starred. As in the 3-O case, one can correlate a high accuracy to a specific loop combination. In this case, the one such combination is de.

## 5. Discussion

A wide variety of techniques for modeling proteins have been explored in the literature, including features based on primary, secondary, and tertiary structure, as well as aspects of proteins such as locations within protein interaction networks or phylogenetic characteristics. In turn, these models have been used in a large variety of classification techniques. Previous graphical models of proteins have generally targeted secondary or tertiary structure [25, 45, 74]. However, graphical models of residues have also been explored in [75]. Often, this has led to topological characteristics that have informed clustering, *k*NN, and similar distance-based classification techniques. Some strategies have also modeled primary structure as time series. [58].

Like traditional sequence alignment, this approach attempts to model the varying lengths inherent in primary structure directly. In contrast, other models typically handle that structural aspect indirectly based on the frequency of residues or residue classes. While SVMs have been used extensively for protein classification, BNNs appear to have been used less extensively on protein classification problems [76–78]. Moreover, most protein classification efforts have been directed to higher level classifications than the functional differences modeled in this work. Further, the authors are aware of only one previous machine learning effort specifically on flavonoid UGT classification [25].

The approach taken here has been successful on the 3-O problem. DTW distances based on the residue models and key loop regions have improved on classification based on traditional sequence alignments. The DTW SVM and BNN techniques correctly identified UGTs misclassified by sequence alignment without introducing offsetting errors of their own. The performance of some loop-index-specific 3NN classifiers was quite good on the 3-O problem, and better than the others on the 7-O problem. The evident correlation between high performing loop-index combinations and misclassified UGTs in the DTW 3NN classifiers is a striking finding.

The approach was not as successful on the 7-O problem. However, there were only 6 positive examples of 7-O to work with and there are strong biochemical reasons for expecting a greater variety of 7-O UGTs than 3-O UGTs [26]. Thus, the limited size of the data set might prove too costly for a good estimate of the model's adequacy.

In addition to the difficulty of estimating generalization error on the 7-O problem due to data set size, other significant limitations should be noted. The accepted class boundaries may be noisy. This should influence the interpretation of the improvements on the 3-O and the 7-O problems. Different hold out sets were used for the BNNs. Hold out set selection was somewhat ad hoc, exhibiting dependencies such as a single set containing UGT characterized by the same methods in the same paper, or the placement of half the 7-O UGTs in a single set. Finally, the uncertainty involved in judging MCMC sampling to have reached equilibrium is another source for reservation.

Nonetheless, the results suggest that the methodological principal has merit. Members of a set of proteins that are similar at the primary and tertiary levels, and for which detailed knowledge of conformation is frequently unavailable to inform modeling efforts, have been successfully classified by fine functional distinctions by modeling primary structure directly in graphical terms and applying several machine learning techniques to features based on subsequences of biochemically informed interest.

The flexibility and insights provided by time series distance models looks especially promising. On the one hand, they allow for directly modeling sequences. On the other hand, they permit a wide variety of learning techniques based on the notion of distances. Additionally, the general scheme is independent of the indices used, which could be varied from case to case. Finally, the computational complexity of time series distances is comparable to sequence alignment, although no match for high throughput heuristics such as BLAST. Potential applications include related problems such as UGT acceptor specificity and analogous fine functional classification problems faced in the study of different proteins.

Future work might enhance the model for primary structure. Other work could focus on the successful 3-O UGT models by investigating classifier performance after selective editing of the signatures. This *in silica* mutagenic approach, so to speak, might combine an analysis of the loop-specific 3NN results with biochemical expertise to inform the signature or loop region edits. Such an effort could conceivably assist in planning genuine mutagenic studies. A technique for rapidly obtaining covering subsequences would facilitate applying the BNN approach to other problems or including a larger data set in the problem investigated here. It would also be interesting to see if padding loop regions with “blank” residues yielding zero index values to obtain uniform length feature vectors could avoid the alignment cost in extending the BNN approach to new data. It is possible that a leave-one-out approach on the 7-O classification effort might yield more accurate results. Additionally, it would be useful to compare the BNN performance with standard ANNs on the 3-O and 7-O problems. Of course, boosting the data set would be valuable for all the methods explored. A fuller analysis of the loop-index-specific results seems indicated, such as an investigation of the mutual information among loops and indices. Finally, other similar biological problems could be studied.

## Figures and Tables

**Table 1:** 23 UGTs with experimentally determined regioselectivity. Legend: ID: numeric identifier for this study; Label: unique abbreviated description; Acceptor: substrate's flavonoid subclass; Organism: enzyme's native organism; Accession: sequence database ID; Class: experimentally determined regioselectivity.

ID	Label	Acceptor	Organism	Accession	Class
1	CsF3GT	Flavonol	* Citrus sinensis *	AAS00612	3
2	RhA53GT	Anthocyanidin	* Rosa hybrida *	BAD99560.1	5, 3
3	VvGT	Anthocyanidin/Flavonol	* Vitis vinifera *	O22304	3
4	IhA3GT	Anthocyanidin	* Iris hollandica *	Q5KTF3	3
5	DcC2′GT	Chalcone	* Dianthus caryophyllus *	Q60FE8	2′
6	DcF3GT1	Anthocyanidin/Flavonol	* Dianthus caryophyllus *	Q60FF0	3
7	DcF3GT2	Anthocyanidin/Flavonol	* Dianthus caryophyllus *	Q60FF2	3
8	GtA3GT	Anthocyanidin/Flavonol	* Gentiana triflora *	Q96493	3
9	CuLGT	Limonoid	* Citrus unshiu *	Q9MB73	NA
10	DbB5GT	Flavone/Flavonol*	* Dorotheanthus bellidiformis *	Q9SMG6	4′
11	SbF7GT	Flavone	* Scutellaria baicalensis *	Q9SXF2	7
12	VhA5GT	Anthocyandin	* Verbena hybrida *	Q9ZR25	5
13	PfA5GT	Anthocyandin	* Perilla frutescens *	Q9ZR27	5
14	At7GT	Flavanone	* Arabidopsis thaliana *	NP_567955	7
15	BvF4′7GT	Flavone/Flavonol	* Beta vulgaris *	AAS94329.1	4′, 7
16	BvF37GT	Flavonol	* Beta vulgaris *	AAS94330.1	3, 7
17	GtA3′GT	Anthocyandin	* Gentiana triflora *	BAC54092	3′
18	GmF7GT	Isoflavone	* Glycine max *	BAF64416	7
19	NtF7GT	Flavonol	* Nicotiana tabacum *	BAB88935.1	7
20	OsF3GT	Flavonol	* Oryza sativa *	NP_001044170	3
21	PhA5GT	Anthocyandin	* Petunia hybrida *	BAA89009.1	5
22	PhA3GT	Anthocyandin	* Petunia hybrida *	BAA89008.1	3
23	UGT71G1	Flavonol*	* Medicago truncatula *	Q5IFH7	3′

**Table 2:** Top Loop-Index-Specific 3NN 3-O Results.

Indices	Missed by bdg	Missed by be	Missed by d
12345	2, 16, 20	5*, 18*	2, 5*, 20
2345	2, 5*, 16, 20	5*, 7, 16, 18*	2, 5*, 20
1345	2, 16, 20	5*, 9*, 14*, 18*	2, 5*, 20
1245	2, 5*, 16, 20, 23*	5*, 18*	2, 5*, 20
1235	2, 16, 20	5*, 18*	2, 5*, 20
1234	2, 16, 20	5*, 18*	2, 5*, 20
345	2, 16, 20	5*, 18*	2, 5*, 20
245	2, 5*, 16, 20, 23*	5*, 7, 16, 18*	2, 5*, 20
235	2, 16, 20	5*, 18*	2, 5*, 20
234	2, 16, 20	5*, 7, 16, 18*	2, 5*, 20
145	2, 5*, 16, 20, 23*	5*, 9*, 14*, 17, 18*	2, 5*, 10*, 15*, 16, 20
135	2, 16, 20	5*, 9*, 14*, 18*	2, 5*, 20
134	2, 16, 18*, 20	5*, 9*, 14*, 18*	2, 5*, 20
125	2, 5*, 16, 20, 23*	5*, 17, 1*8	2, 5*, 20
124	2, 5*, 16, 20, 23*	5*, 18*	2, 5*, 20
123	2, 16, 20	5*, 18*	2, 5*, 20
45	2, 5*, 20, 23*	7, 18*	2, 5*, 20
35	2, 16, 18*, 20	5*, 9*, 18*	2, 5*, 18*, 20
34	2, 9*, 16, 20	5*, 7, 9*, 14, 18*	2, 5*, 20
25	2, 5*, 20	7, 18*	2, 5*, 20, 23*
24	2, 5*, 9*, 16, 20, 23*	5*, 7, 16, 18*	2, 5*, 20
23	2, 16, 20	5*,18*	2,5*, 20
15	2, 16, 20, 23*	5*, 9*, 14*, 17*, 18*, 23*	2, 5*, 21*
14	2, 5*, 16, 20, 23*	5*, 7, 9*, 14*, 17*, 18*	2, 5*, 20
13	2, 16, 20	5*, 7, 9*, 10, 14, 17*, 18*	2, 5*, 20
12	2, 5*, 16, 20, 23*	5*, 18	2, 5*, 20
5	2, 5*, 18*, 21*	9*, 17, 18*, 23*	2,9*, 18*, 21*, 23
4	2, 5*, 9*, 14*, 16, 18*, 20, 23	7, 14*, 16, 20	2, 5*, 20
3	2, 9*, 14*, 16, 18*, 20	5*, 9*, 14*, 18*, 23*	2, 5*, 18*, 20
2	2, 5*, 20	5*, 7, 15*, 16, 18*	2, 5*, 20
1	2, 11*, 16, 20, 23*	5*, 9*, 11*, 12*, 14*, 18*, 19*	2, 5*, 20

**Table 3:** Loop-Index- Specific 3NN 7-O Results.

Loop-Index	7-O Errors
a2	10*, 16, 18
e145	1*, 11, 19
de14	11, 17*, 19
de45	10*, 16, 19
de134	11, 17*, 19
de245	10*, 11, 19
de1245	10*, 11, 19
de1345	11, 17*, 19
eg14	11, 18, 19
eg125	11, 18, 19
